# Risk of eating disorders in international adoptees: a cohort study using Swedish national population registers

**DOI:** 10.1017/S2045796020000451

**Published:** 2020-05-26

**Authors:** M. Strand, R. Zhang, L. M. Thornton, A. Birgegård, B. M. D'Onofrio, C. M. Bulik

**Affiliations:** 1 Stockholm Centre for Eating Disorders, Wollmar Yxkullsgatan 27B, 118 50 Stockholm, Sweden; 2 Department of Clinical Neuroscience, Centre for Psychiatry Research, Karolinska Institutet, & Stockholm Health Care Services, Stockholm County Council, 171 77 Stockholm, Sweden; 3 Department of Medical Epidemiology and Biostatistics, Karolinska Institutet, Stockholm, Sweden; 4 Department of Psychiatry, University of North Carolina School of Medicine, Chapel Hill, NC, USA; 5 Department of Nutrition, Gillings School of Global Public Health, University of North Carolina, Chapel Hill, NC, USA; 6 Department of Psychological and Brain Sciences, Indiana University, Bloomington, IN, USA

**Keywords:** Epidemiology, mental health, mood disorders unipolar, obsessive-compulsive disorder, other disorders

## Abstract

**Aims:**

Compared to the general population, adoptees are more often referred to specialist psychiatric treatment, exhibit increased risk of suicide and display more symptoms of attention-deficit/hyperactivity-disorder. However, little is known about the impact of being an adoptee on the risk of developing an eating disorder. The aim of the present study was to assess whether international adoptees have a higher risk for eating disorders than native Swedes.

**Methods:**

In the present retrospective cohort study, data from the Swedish total population registers on individuals born between 1979 and 2005 were used to assess whether international adoptees residing in Sweden (*n* = 25 287) have a higher risk for anorexia nervosa (AN) and other eating disorders (OED) than non-adoptees with Swedish-born parents from the general population (*n* = 2 046 835). The patterns of these results were compared to those for major depressive disorder (MDD), obsessive-compulsive disorder (OCD), and anxiety disorders to determine whether any observed effects were unique to eating disorders or reflected a more general impact on mental health outcomes.

**Results:**

A survival analysis adjusting for relevant demographic covariates revealed an elevated risk of all examined psychiatric disorders in international adoptees: hazard ratios (95% confidence intervals) are 1.21 (1.04–1.41) for AN, 1.60 (1.44–1.79) for OED, 1.90 (1.81–2.00) for MDD, 1.25 (1.09–1.44) for OCD, and 1.69 (1.60–1.78) for anxiety disorders.

**Conclusions:**

Elevated risk of eating disorders as well as of MDD, OCD, and anxiety disorders was found in international adoptees. A parallel pattern between AN and OCD was observed, which both display less elevated rates than the other diagnoses. A considerable number of biological, environmental, and societal factors have been suggested to explain the observed differences in mental health between adoptees and non-adoptees, but they remain primarily theoretical.

## Introduction

Adoption is a global phenomenon that touches the lives of many families worldwide. Domestic adoption of children whose biological parents are deceased or cannot provide for them has been practised in human societies since ancient times. In contrast, international adoption – i.e., when children are placed for adoption outside their country of birth – is a more recent phenomenon, emerging after World War II and evolving on a broader scale in the aftermath of the Korean War (Selman, 2012). From the 1960s and onwards, international adoption has become the dominant form of adoption in many Western countries, as fewer children have been put up for national or domestic adoption; this shift is in part due to increased availability of contraceptives, the introduction of family planning services, and a less stigmatized view of single parenthood and children born out of wedlock (Palacios *et al*., 2019). It has been estimated that over 1 million children have been adopted across national borders since the end of World War II (Grotevant and McDermott, 2014), underscoring the importance of attending to their well-being.

However, marked differences exist between countries. In some parts of the world, predominantly in poorer countries, adoption involves sending children abroad, whereas elsewhere, predominantly in wealthier countries, adoption primarily involves the receipt of children from these other parts of the world (Neil and Miller Wrobel, 2012; Grotevant and McDermott, 2014). In the USA, national adoption (e.g., the subsequent adoption of children placed in foster homes) still makes up a major share of adoption cases (Palacios *et al*., 2019), whereas, in a country such as Sweden, a large majority of adoption cases are international (excluding adoption within the family, such as when step-parents adopt the biological children of their spouse) (Statistics Sweden, 2018). Unlike in earlier decades, due to socioeconomic development and altered policies in many origin countries, a surplus of prospective adoptive parents currently exists. As countries such as India and South Korea have seen the emergence of large middle-class populations, the legitimacy of sending children abroad for adoption has been questioned (Triseliotis, 2000; Wiley, 2017) and accompanied by a parallel move towards domestic adoption in these countries (Selman, 2012; Palacios *et al*., 2019). Since the 1990s and during the later years covered in the present study, China and Russia have been the leading countries in sending children abroad for adoption (Selman, 2012).

Research on the mental health of adoptees has revealed concerning tendencies on a group level. Compared to the general population, adoptees are more often referred to specialist outpatient and inpatient psychiatric treatment (Hjern *et al*., 2002; Lindblad *et al*., 2003; Juffer and van IJzendoorn, 2005), exhibit increased risk of suicide attempts and suicide (Hjern *et al*., 2002; Hjern *et al*., 2004; von Borczyskowski *et al*., 2006), and more often display symptoms of attention-deficit/hyperactivity-disorder (ADHD) (Lindblad *et al*., 2010) and externalizing behavioural problems (Juffer and van IJzendoorn, 2005; Askeland *et al*., 2017; Barroso *et al*., 2017) that may require residential care during adolescence (Elmund *et al*., 2007). Moreover, adoptees are more often unemployed and dependent on social welfare, are less likely to be in a relationship and to have children, and when they do have children, they are more likely to be single parents (Lindblad *et al*., 2003; Tieman *et al*., 2006). These findings become even more striking after adjusting for socioeconomic variables; in most places, a majority of adoptees have been raised in middle- or high-income adoptive families, who usually display better health outcomes than the larger population (Hjern *et al*., 2002, 2004; von Borczyskowski *et al*., 2006).

However, research in the field is heterogeneous, rendering comparisons difficult across studies. For example, national and international adoptee samples are not necessarily comparable in terms of early adverse experiences. Due to the non-disclosure of information about the biological family, it is usually very difficult to assess the pre-adoption experiences of international adoptees (Verhulst *et al*., 1992; Boer *et al*., 1994). Adoptee samples also vary in other aspects. A large longitudinal study of Romanian children adopted into British families in the post-Ceaușescu era has highlighted the impact of early institutional deprivation in this group (Rutter and Sonuga-Barke, 2010; Kumsta *et al*., 2012; Rutter *et al*., 2012). Adoptees in this particular sample, however, were arguably subjected to comparably high levels of pre-adoption adverse experiences and the findings may not be representative for international adoptee groups in general. Rather than focusing narrowly on adoption status, it may be more meaningful to view the adoptee population as a heterogeneous group of individuals that may, for instance, have been exposed to early traumatic events to a greater extent than the general population (Lindblad *et al*., 2003). Higher age at adoption has long been considered a risk factor for mental health problems in adoptees (Palacios *et al*., 2019), but rather than the age per se, the central aspect may be the number of adverse pre-adoption experiences, where higher age could imply greater exposure (Barroso *et al*., 2017). Additionally, fluctuating developmental sensitivity across the childhood years could implicate age of placement as an important factor (Grotevant and McDermott, 2014).

A considerable number of genetic, biological, environmental, and societal mechanisms (as well as interactions among them) have been suggested to explain the observed differences in mental health between adoptees and non-adoptees. For example, heritable psychopathology in biological parents (Manhica *et al*., 2016) and early puberty (Bimmel *et al*., 2003; Ekeus *et al*., 2009) have been implicated as potential underlying mechanisms. Moreover, a number of pre-adoption factors, such as adverse environmental impact during pregnancy (Lindblad *et al*., 2010), maltreatment and neglect at adoption facilities (Juffer and van IJzendoorn, 2005; Palacios *et al*., 2019), and separation from early attachment figures (Palacios *et al*., 2019) could hypothetically contribute to the observed patterns.

Factors associated with the adoptive family and the adoptee status have also been highlighted, such as stress reactions upon disclosure of adoption history (Palacios *et al*., 2019) and problems in identity formation (Juffer and van IJzendoorn, 2005; Askeland *et al*., 2017; Palacios *et al*., 2019). Furthermore, a referral bias due to adoptive parents' socioeconomic resourcefulness (Lindblad *et al*., 2010; Barroso *et al*., 2017) and attentiveness to problematic behaviours in their children (Askeland *et al*., 2017) may exist.

Finally, factors associated with navigating racial differences, such as feelings of not belonging (Juffer and van IJzendoorn, 2005), discrimination, and racism (Askeland *et al*., 2017; Palacios *et al*., 2019) could affect the mental health of international adoptees. For example, some adoptees report that adoptive parents' ‘colourblindness’ may leave them having to manage racialised societal stereotypes solely on their own (Wiley, 2017; Palacios *et al*., 2019).

Eating disorders, such as anorexia nervosa (AN), bulimia nervosa (BN), and binge-eating disorder (BED), are responsible for a considerable disease burden on a global scale due to both disability and mortality (Erskine *et al*., 2016). AN is characterized by restriction of energy intake leading to significantly low body weight, an intense fear of weight gain, and a disturbed experience of one's own body weight or shape. BN is characterized by recurrent episodes of binge eating, inappropriate compensatory behaviours to prevent weight gain (such as vomiting, laxative use, or excessive exercise), and an overemphasis on weight or shape in self-evaluation. BED is characterized by recurrent episodes of binge eating without compensatory behaviours. Eating disorders are associated with a multitude of potential medical complications, such as cardiac dysfunction, reduction of bone density, and severe electrolyte imbalances, and psychiatric comorbidity has been described as the rule rather than the exception (Treasure *et al*., 2010). Individuals with an eating disorder display significantly elevated mortality rates; the standardized mortality ratio in AN, estimated at 5.9, is much higher than in any other psychiatric disorder (Arcelus *et al*., 2011).

Little is known about any potential impact of being an adoptee on the risk of developing an eating disorder. Building upon an earlier case study (Fry and Crisp, 1989), a British study found that 3.8% of patients with AN or BN referred to the Maudsley Hospital in London between 1975 and 1985 were adoptees, compared to an adoptee rate of 1.5% in the corresponding age span in the general population (Holden, 1991). In contrast, a Swedish register study evaluating 11 320 international adoptees showed that, whereas the risks of suicide and of being admitted for a psychiatric disorder were increased in this group, prevalence of AN did not differ between adoptees and the general population (Hjern *et al*., 2002). This study, however, was based on data from registered hospital discharge diagnoses, which are unlikely to accurately reflect the actual prevalence of eating disorders because most of these patients are treated in outpatient settings. A Dutch study, in which 1 484 international adoptees residing in the Netherlands were assessed with diagnostic interviews, similarly found no differences in eating disorder prevalence between adoptees and non-adoptee controls (Tieman *et al*., 2005). However, this study was inadequately powered to be able to detect meaningful differences in disorders of relatively low prevalence, such as eating disorders. In a Swedish community study on disordered eating in a non-clinical population utilising survey data comprising almost 115 000 participants, international adoptee women displayed significantly higher levels of self-induced vomiting, loss-of-control eating, food preoccupation, underweight, and drive for thinness compared to non-adoptee women, albeit with small effect sizes (Strand *et al*., 2019). In general, the fact that adoptees constitute a small minority of the population can make it difficult to detect rare presentations in this subgroup even in comparably large survey samples. Notably, Sweden currently has the largest per capita proportion of international adoptees in the world, at 0.56% of the population (Statistics Sweden, 2014).

Importantly, when studying mental health conditions such as eating disorders in international adoptees, it is vital to try to ascertain whether an observed elevated risk is indeed specific for eating disorders or if it mirrors broader non-specific effects on mental health in this group. One way of exploring these issues is to compare the risk of eating disorders with those of other common psychiatric disorders, such as major depressive disorder (MDD), obsessive-compulsive disorder (OCD), and anxiety disorders in the adoptees. In the present retrospective cohort study, we utilise data from the Swedish total population registers to assess whether international adoptees have a higher risk for eating disorders than native Swedes. We also compare the patterns of these results to those for MDD, OCD, and anxiety disorders to determine if any observed effects are unique to eating disorders or reflect a more general pattern of impact of adoption on subsequent mental health. The study cohort consists of 25 287 international adoptees residing in Sweden and 2 046 835 non-adoptees with Swedish-born parents from the general population; thus, we anticipate achieving adequate statistical power to be able to detect clinically relevant differences in the risk of developing eating disorders.

## Methods

### Study design and materials

In this cohort study, the exposed group was defined as international adoptees in Sweden; more specifically, individuals in the international adoptee group were born abroad between 1979 and 2005 and adopted before 8 years of age by Swedish-born parents residing in Sweden. This group, in turn, was compared to a referent population of individuals born between 1979 and 2005 by Swedish-born biological parents and residing in Sweden during the follow-up period. Thus, national adoptees and international adoptees raised by adoptive parents who were born abroad themselves were excluded from the analysis, due to the low numbers of national adoptees in the population (*n* = 105) as well as differences in risk of eating disorders in immigrant populations in general (Mustelin *et al*., 2017). The study makes use of the high-quality nationwide registers maintained by the Swedish government, which covers the Swedish population in its entirety using the unique personal identification number given to all Swedish citizens (Ludvigsson *et al*., 2016). In Sweden, population registration was computerized in 1967 and in the following year Statistics Sweden used this population register to establish a register system called the Total Population Register (TPR), which contains population and household statistics including birth, death, family relationships, and migration within Sweden as well as to and from other countries (Statistics Sweden, 2017). Furthermore, education level from the Longitudinal integrated database for health insurance and labour market studies (LISA) at Statistics Sweden and diagnosis from the National Patient Register (NPR), the Cause of Death Register (CDR) at the Swedish National Board of Health and Welfare, and the national eating disorder quality registers Riksät (Swedish Association of Local Authorities and Regions, 2007) and Stepwise (Birgegård *et al*., 2010) were used. The use of these data has been approved by the regional ethical review board in Stockholm, Sweden. In reporting our findings, we have adhered to the STROBE statement on improving the quality of reporting of observational studies (von Elm *et al*., 2007).

### Study population

The study cohort included individuals born between January 1, 1979 and December 31, 2005 (3 424 933 individuals) registered in the Swedish TPR with the following sequential exclusions: stillbirths and congenital malformations (*n* = 103 245), death before 8th birthday (*n* = 12 882), emigration before 8th birthday (*n* = 106 065), no complete parental information (biological/adoptive) (*n* = 433 876), no birth country information (*n* = 188), parents born outside of Sweden (*n* = 695 642), adoptees born in Sweden (*n* = 46), and immigration after 8th birthday (*n* = 866), yielding a final sample of 2 072 123 individuals. In total, the study cohort consists of 25 287 international adoptees residing in Sweden and 2 046 835 non-adoptees with Swedish-born parents. We followed the study population until December 31, 2013, when the youngest individuals were 8 years old and the oldest individuals were 34 years old.

### Exposure and covariates

For the purposes of this study, international adoptees were defined as individuals born abroad between 1979 and 2005 and adopted before 8 years of age by Swedish-born parents residing in Sweden. Information on sex and birth year, as well as birth years of adoptive parents, was obtained from the TPR. Date of adoption was defined as the immigration date of the adoptee. Data on maternal education level, defined as the adoptive mothers' years of attained education in 2013, categorized as ⩽9 years (i.e., less than the Swedish mandatory minimum level of 9 years of school), 10–12 years (i.e., some upper secondary education), 13–14 years, or 15+ years, were obtained from the LISA database. Because the Swedish population registers only started to record education level in 1990, data on parental education level at the time of birth/adoption were not available for all individuals in our cohort. Instead, data on maternal education level at the end of follow-up were used, in accordance with a previous study on ADHD in international adoptees in which a strong correlation between maternal and paternal education levels was also found (Lindblad *et al*., 2010). It should also be noted that the handling of data on the ethnicity or race of individuals is generally not permitted by Swedish law. Data on the geographical origin on the regional level for the international adoptees in our cohort are provided as online Supplementary material; however, data on individual countries of origin were not available.

### Study variables

We defined and analysed eating disorders grouped in two ways based on lifetime diagnoses according to the International Statistical Classification of Diseases and Related Health Problems (ICD) and/or the Diagnostic and Statistical Manual of Mental Disorders (DSM) in the NPR, CDR, and the eating disorder quality registers before December 31, 2013. For the specific ICD diagnostic codes used, see Table 1. In the eating disorder quality registers, AN was defined as meeting DSM-IV-TR criteria for AN (307.1) or atypical AN (307.50, criteria 1 and 2) (here, other lifetime eating disorders could be present), and OED was defined as meeting DSM-IV-TR criteria for any eating disorders other than AN (BN (307.51), atypical BN (307.50, criteria 3), or eating disorders not otherwise specified). Both AN and OED were recorded if an individual received both diagnoses. Individuals with MDD, OCD, or anxiety disorder were identified in the NPR and CDR using the ICD diagnostic codes outlined in Table 1.

**Table 1.** ICD-8, ICD-9, and ICD-10 codes from the National Patient Register and the Cause of Death Register

Diagnosis	ICD-8	ICD-9	ICD-10
Anorexia nervosa	–	307B	F50.0, F50.1
Other eating disorder	–	307F	F50.2, F50.3, F50.9
Major depressive disorder	300.4	296B, 300E, 311	F32.0, F32.1, F32.2, F32.3, F32.8, F32.9, F33.0, F33.1, F33.2, F33.3, F33.4, F33.8, F33.9, F34.8, F34.9, F38.0, F38.1, F38.8, F39
Obsessive-compulsive disorder	300.3	300D	F42.0, F42.1, F42.2, F42.8, F42.9
Any anxiety disorder	300.0, 300.2	300A, 300C	F40.0, F40.1, F40.2, F40.8, F40.9, F41.0, F41.1, F41.2, F41.3, F41.8, F41.9

### Statistical analysis

To assess whether the risk of eating disorders and other selected psychiatric disorders differ between international adoptees in Sweden compared with native Swedes, hazard ratios with 95% confidence intervals for each eating disorder group (i.e., AN and OED) and other psychiatric disorders (i.e., MDD, OCD, and anxiety disorders) were estimated from Cox proportional hazard models. Adoption status was treated as time-varying exposure. Individuals were followed from their 8th birthday until the onset of the selected psychiatric disorders, death, emigration from Sweden, or the end of the follow-up period (December 31, 2013), whichever came first. Both crude and adjusted models are reported. In the adjusted model, we adjusted for sex, birth year (for both adoptee and adoptive parents), and maternal education level. We used a cluster-robust (sandwich) estimator for standard error calculation, where clusters were identified via family identification numbers to control for intra-familial correlation within the data. Data management was performed using SAS, version 9.4. (SAS Institute Inc., 2013). Statistical analyses were performed using R, version 3.4.3 and the ‘survival’ R package specifically (Therneau and Lumley, 2019).

## Results

Descriptive characteristics of the international adoptee and non-adoptee populations regarding sex, maternal education, and prevalence of eating disorders and other selected psychiatric disorders (including basic stratification based on sex) are provided in Table 2. Of the 25 287 identified international adoptees, 53.69% were female compared with 48.76% of the 2 046 835 native Swedes. The adoptive mothers tended to have more education than mothers of native Swedes.

**Table 2.** Descriptive characteristics and psychiatric disorder prevalence stratified by adoptive status and sex

	International adoptees (%)	Non-adoptees (%)
	*n* = 25 287	*n* = 2 046 835
Characteristic		
Sex		
Male	11 711 (46.31)	1 048 815 (51.24)
Female	13 576 (53.69)	998 020 (48.76)
Maternal education		
⩽ 9 Years	1 692 (6.69)	177 806 (8.69)
10–12	9 146 (36.17)	985 426 (48.14)
13–14	13 024 (51.50)	820 575 (40.09)
15 +	383 (1.51)	17 176 (0.84)
Missing data	1 042 (4.12)	45 853 (2.24)
Psychiatric disorders		
AN	181 (0.72)	10 435 (0.51)
Male	10 (0.09)	609 (0.06)
Female	171 (1.23)	9 826 (0.98)
OED	364 (1.44)	16 809 (0.82)
Male	30 (0.26)	1 064 (0.10)
Female	334 (2.46)	15 745 (1.58)
MDD	1 880 (6.63)	80 962 (3.96)
Male	686 (5.86)	29 647 (2.83)
Female	1 194 (8.79)	51 315 (5.14)
OCD	225 (0.89)	12 591 (0.62)
Male	105 (0.90)	5 316 (0.51)
Female	120 (0.88)	7 275 (0.73)
Anxiety disorders	1 676 (7.43)	82 551 (4.03)
Male	579 (4.94)	29 782 (2.84)
Female	1 097 (8.08)	52 769 (5.29)

AN, anorexia nervosa; OED, other eating disorder; MDD, major depressive disorder; OCD, obsessive-compulsive disorder.

In Table 3, hazard ratios of eating disorders and other selected psychiatric disorders are presented. Here, the crude model is not adjusted for any covariates, whereas the adjusted model is adjusted for sex, individual's birth year, maternal birth year, paternal birth year, and maternal education level. In both the crude and adjusted models, risk of all psychiatric disorders examined was elevated in international adoptees. In the case of MDD and anxiety disorders, adjusting for covariates increased hazard ratios with point estimates increasing and confidence intervals not overlapping. AN and OCD yielded the lowest estimates and were unaffected by adjustment for covariates. A Kaplan–Meier curve outlining the absolute risks over time is available as online Supplementary material.

**Table 3.** Hazard ratios of psychiatric disorders in international adoptees compared to non-adoptees

	Crude model^a^	Adjusted model^a^
	HR (95% CI)	HR (95% CI)
AN	1.27 (1.09–1.47)	1.21 (1.04–1.41)
OED	1.54 (1.39–1.71)	1.60 (1.44–1.79)
MDD	1.61 (1.54–1.69)	1.90 (1.81–2.00)
OCD	1.24 (1.09–1.42)	1.25 (1.09–1.44)
Anxiety disorders	1.39 (1.32–1.46)	1.69 (1.60–1.78)

HR, hazard ratio; CI, confidence interval; AN, anorexia nervosa; OED, other eating disorder; MDD, major depressive disorder; OCD, obsessive-compulsive disorder.

a Crude model is unadjusted and the adjusted model is adjusted for sex, individual's birth year, maternal birth year, paternal birth year, and maternal education level.

## Discussion

This cohort study, using the Swedish national population registers, found that international adoptees are at an elevated risk for AN and OED than are non-adoptees, both in the crude and the adjusted model. However, the effect is not specific; international adoptees are also at elevated risk for the comparison of psychiatric disorders of MDD, anxiety disorders, and OCD. Adjusting for relevant demographic covariates including sex, child's birth year, maternal birth year, paternal birth year, and maternal education level had differential effects across outcomes, with the risk being slightly attenuated in AN, fairly stable for OED and OCD, and elevated in MDD and anxiety disorders.

As described above, a considerable number of genetic, biological, environmental, and societal mechanisms have been suggested to explain the observed differences in mental health between adoptees and non-adoptees, some of which could also contribute to the elevated risk of AN and OED in the adoptee group. However, they remain primarily theoretical as little empirical evidence exists to clarify the nature and extent of influence of these factors. With regard to the observed elevated risk of eating disorders in this group, experiences of having a physical appearance that differs from that of the majority population could hypothetically give rise to body image concerns similar to those reported in immigrant groups (Perez *et al*., 2002). However, the existing literature on body dissatisfaction in ethnic minority groups is ambiguous (Kimber *et al*., 2014; Doris *et al*., 2015).

Another factor that could hypothetically contribute to the observed patterns is early malnutrition. Many international adoptees have been exposed to malnutrition in the first months or years after birth and/or in utero which may, for instance, result in a delayed growth in terms of height, weight, and head circumference (Van IJzendoorn *et al*., 2007). Studies of the effects of historical events such as the Dutch Hunger Winter 1944–45 or the Chinese Great Leap Forward Famine 1959–61 on physical and mental health have shown an association between prenatal famine and adult body size, diabetes mellitus, and schizophrenia (Lumey *et al*., 2011). There are also indications (including studies on historical data from Överkalix in northern Sweden) that these effects may be epigenetically transferred across generations (Kaati *et al*., 2007; van den Berg and Pinger, 2016). Early malnutrition, as well as other early-life adversities, can induce long-term alterations in the metabolic and neuroendocrine systems that increase vulnerability to stress and obesity risk throughout life (Sawaya *et al*., 2004; Yam *et al*., 2015); such mechanisms could in theory also be involved in the development of eating disorders.

Notably, although the risk was elevated in all psychiatric disorders in the adoptee group, the hazard ratios of AN and OCD were numerically lower than those of OED, MDD, and anxiety disorders. Previous research on the genetics of AN and OCD has revealed a high degree of genetic sharing (Cederlöf *et al*., 2015), which may contribute to the parallel patterns between the two diagnoses observed here. Additionally, AN and OCD may be more homogeneous and biologically driven, thereby less reactive to environmental factors associated with adoption that could influence disorder expression (Pauls *et al*., 2014; Cederlöf *et al*., 2015; Larsen *et al*., 2017; Watson *et al*., 2019). In contrast, the OED numbers more closely resemble those of MDD and anxiety disorders. The observed elevated risk in MDD and anxiety disorders after adjustment may reflect the fact that the expression of genetic risk for these disorders may be more influenced by environmental factors (Sullivan *et al*., 2000; Hettema *et al*., 2001). For example, it could be hypothesized that adoptive parents on a group level provide a relatively stable family environment due to their generally older age and the usually highly selective adoption process and that this is reflected in the difference between the crude and adjusted models. Naturally, our data do not allow for any definitive conclusions regarding the interrelations between eating disorders and other psychiatric disorders in international adoptees and the discussion above should, therefore, be seen as hypothetical.

These findings build on previous studies that have shown that adoptees more often present for specialist psychiatric treatment (Hjern *et al*., 2002; Lindblad *et al*., 2003; Juffer and van IJzendoorn, 2005), exhibit increased risk of suicide attempts and suicide (Hjern *et al*., 2002, 2004; von Borczyskowski *et al*., 2006), and display more symptoms of ADHD (Lindblad *et al*., 2010) and externalizing behavioural problems (Juffer and van IJzendoorn, 2005; Askeland *et al*., 2017; Barroso *et al*., 2017). Whereas previous studies on eating disorders in adoptees have been inconclusive due to methodological shortcomings, such as inadequate statistical power, here we overcome this barrier by using nationwide high-quality population registers. The present study is the largest study on eating disorders in international adoptees to this date, using population registers appropriately chosen so as to accurately reflect the true prevalence of detected eating disorders in this population.

Nonetheless, the heterogeneous nature of the adoptee population poses challenges to generalisability. It is, for example, known that adoptees with origins in South Korea tend to fare better in psychological terms in comparison with adoptees with a Latin American origin (Lindblad *et al*., 2003; Elmund *et al*., 2007; Odenstad *et al*., 2008). This may be due to the fact that in South Korea, the factors causing a child to be put up for adoption have mostly been social, such as a prevailing stigma of single motherhood (Boer *et al*., 1994), and South Korean adoption facilities have had a relatively high standard of care (Kim, 1995; Kim *et al*., 1999; Odenstad *et al*., 2008). Similar favourable patterns in terms of behavioural adjustment and academic performance have been observed in adoptees of Chinese origin in North America (Tan and Marfo, 2006; Cohen and Farnia, 2010; Tan *et al*., 2015). In Latin America, in contrast, the reasons behind adoption are more often related to poverty (Boer *et al*., 1994), implying that adoptees born in Latin America could have been subjected to more adverse pre-adoption experiences. In Sweden, a large share of international adoptees have been born in South Korea or, more recently, in China (Statistics Sweden, 2014); this fact could have resulted in more beneficial outcomes for adoptees in our sample, underestimating the actual prevalence of eating disorders in international adoptees in a broader setting.

A strength of the present study is that it relies on data from well-established high-quality Swedish population registers (Ludvigsson *et al*., 2011, 2016). The diagnostic validity in these registers has been shown to be high for psychiatric disorders such as schizophrenia (Dalman *et al*., 2002), bipolar disorder (Sellgren *et al*., 2011), and OCD (Rück *et al*., 2015). Likewise, the methods for data collection for the national eating disorder quality registers Riksät and Stepwise have been found to be valid and reliable (Birgegård *et al*., 2010; Emilsson *et al*., 2015). Even so, we cannot exclude the possibility of some differential misclassification in the register data; international adoptees may be either underrepresented in the diagnostic registers due to, for example, foreign-born individuals presenting or being assessed differently by clinicians, or overrepresented due to a potential referral bias associated with adoptive parents' socioeconomic resourcefulness.

Due to the fact that international adoption was relatively uncommon before the 1960s, the adoptee group tends to be younger on average than the population at large, which can make comparisons between groups difficult. In the present study, this problem is avoided because only patients born between 1979 and 2005 are included. Furthermore, the results of the survival analysis were adjusted for birth year. However, this also means that individuals diagnosed with an eating disorder in their late 30s or later are not captured by the analysis. Moreover, it can be noted that the younger individuals in the sample will not yet have passed the main age of risk for eating disorders; however, the fact that the data are right-censored in this regard is taken into account in the survival analysis approach. These limitations are unlikely to have affected our results given the average age of onset of the disorders unless there are substantial differences in the age of onset between groups.

Importantly, in order to be able to examine any effects of early adverse experiences specifically, other research methods and comparison groups (such as non-adopted children with recorded early adverse experiences) would have been required.

In sum, this cohort study using Swedish national population registers reveals that eating disorders, especially eating disorders other than AN, are more prevalent in international adoptees compared to non-adoptees; this finding is nonspecific and is also observed for other psychiatric disorders. An observed parallel pattern between AN and OCD, which both display less elevated rates than the other diagnoses, may be due to shared genetic factors between these two disorders, reflect a more fundamental biological aetiology that is less affected by the environment, or other unmeasured factors. Consensus has not been reached in the literature regarding causal mechanisms for elevated risk for psychiatric disorders in international adoptees and future studies should aim to chart such pathways. Critically, health care providers who care for international adoptees should remain vigilant for psychiatric symptoms in their patients and be made aware of the elevated risk to ensure the health and well-being of the adoptee population.

## Supplementary material

For supplementary material accompanying this paper visit http://dx.doi.org/10.1017/S2045796020000451. click here to view supplementary material

## Data

Data are available from the authors on reasonable request.
