# Supplementary material for: Risk of eating disorders in international adoptees: a cohort study using Swedish national population registers
Source: Epidemiol Psychiatr Sci. 2020 May 26;29:e131. doi: 10.1017/S2045796020000451 (PMC7264708; doi:10.1017/S2045796020000451)

**SUPPLEMENTARY MATERIAL:**

| **Table 4. Geographical origin of international adoptees in the cohort.** | |
| --- | --- |
| **Region of origin** | **Number of individuals** |
| European Union (except Sweden) and Great Britain | 2 049 |
| Europe (except European Union and Great Britain) | 995 |
| Africa | 715 |
| North America | 377 |
| South America | 5 987 |
| Asia and Oceania | 15 141 |
| Soviet Union | 23 |

**Figure 1. Kaplan-Meier curve.**


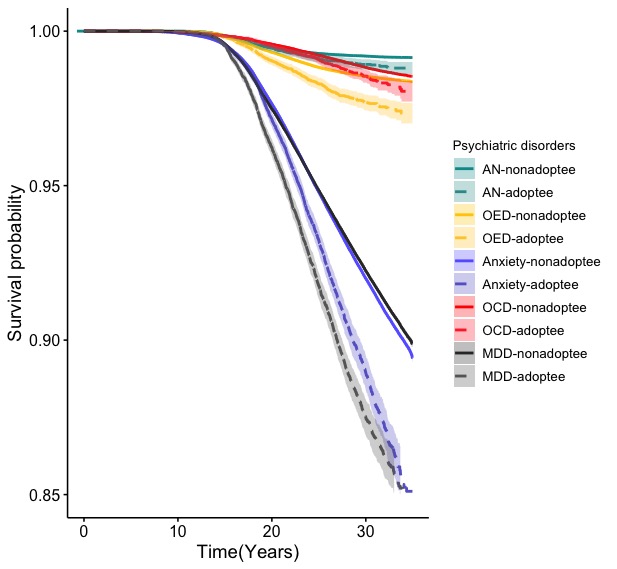

Supplement: Supplementary file 1 [file S2045796020000451sup001.docx]
